# Discrepancies between declared and real practices of continuous renal replacement therapy for septic acute kidney injury in French intensive care units

**DOI:** 10.1016/j.aicoj.2026.100077

**Published:** 2026-05-14

**Authors:** Loïz Stephan, Renaud Prevel, Charline Sazio, Hugo Ilciukas-Miquel, Adrien Auvet, Simon Bodot, Riad Chelha, Valentin Coirier, Xavier Fabre, Aude Garin, Jimmy Garraud, Chloé Gisbert-Mora, Guillaume Grillet, Jan Hayon, Guylaine Labro, Alexis Lambour, Théophile Lancrey-Javal, Eliott Le Basnier, Marc Mikulski, Jonathan Nicolas, Kévin Olry, Fabienne Plouvier, Thierry Seguin, Audrey Tientcheu, Marc Valette, Olivier Guisset, Didier Gruson, Alexandre Boyer, Arthur Orieux

**Affiliations:** a Service de Médecine Intensive Réanimation, CHU de Bordeaux, France; b Univ Bordeaux, Centre de Recherche Cardio-Thoracique de Bordeaux, Inserm U1045, F-33000 Bordeaux, France; c Service de Réanimation - CH de Dax, Dax, France; d Service de Réanimation - CH de Pau, Pau, France; e Service de Réanimation - Hôpital Claude Galien, Quincy-Sous-Sénart, France; f Service de Médecine Intensive Réanimation - CHU de Rennes, Rennes, France; g Service de Réanimation - CH de Roanne, Roanne, France; h Service de Médecine Intensive Réanimation - CH de Dreux, France; i Service de Médecine Intensive Réanimation - CHU de Clermont-Ferrand, Clermont-Ferrand, France; j Service de Réanimation - CH Côte Basque, Bayonne, France; k Service de Réanimation Polyvalente - GHBS site du Scorff, Lorient, France; l Service de Médecine Intensive Réanimation - CHI Poissy Saint-Germain-en-Laye, Poissy, France; m Service de Médecine Intensive Réanimation, GHR Mulhouse et Sud Alsace, Mulhouse, France; n Service de Médecine Intensive Réanimation - CHU de Amiens, Amiens, France; o Service de Médecine Intensive Réanimation - CHU de Nantes, Nantes, France; p Service de Réanimation - CH Le Mans, Le Mans, France; q Service de Réanimation - CHT Gaston-Bourret, Nouméa, France; r Service de Médecine Intensive Réanimation - CHU Rouen, Rouen, France; s Service de Réanimation - CH Émile Durkheim, Épinal, France; t Service de Réanimation - CH Agen Nérac, Agen, France; u Service de Réanimation - CHU de Toulouse, Toulouse, France; v Service de Médecine Intensive Réanimation - CHU de Saint Étienne, Saint Étienne, France; w Service de Réanimation - CHU de Guadeloupe, Les Abymes, France; x Univ Bordeaux, Biology of Cardiovascular Diseases, Inserm UMR 1034, F-33000 Bordeaux, France

**Keywords:** Septic acute kidney injury, Continuous renal replacement therapy, Intensive care unit, Practices

## Abstract

**Background:**

Acute kidney injury (AKI) requiring continuous renal replacement therapy (CRRT) is frequent in the intensive care unit (ICU). While the timing of initiation has been extensively studied, discontinuation remains poorly defined. Current guidelines provide only vague recommendations, and CRRT liberation is mainly guided by clinical judgment.

**Methods:**

We aimed to compare declared and real CRRT weaning practices among intensivists in septic AKI.

We conducted one study with two complementary investigations. Phase 1 was a nationwide survey distributed to 230 French ICUs that explored physician experience, weaning criteria, fluid balance management, urine output thresholds, and urinary biomarker use. A total of 243 intensivists from 87 ICUs responded. Phase 2 was a multicentre retrospective cohort study describing real-world management across 22 ICUs of patients with septic AKI who initiated and were subsequently weaned from CRRT. Weaning was defined as the first discontinuation of CRRT with the intent to stop, regardless of later resumption. We collected clinical, hemodynamic, renal, and fluid data over the 5 days before Day 0, the weaning day.

**Results:**

In survey phase, 93% of respondents cited urine output as the main criterion for discontinuation, with a mean reported threshold of 612 ± 328 mL/day; 74% reassessed daily, and only 3% reported using a protocol. In the retrospective cohort phase (n = 116), markedly lower urine outputs were observed at weaning (median 275 mL [30–967] on D0, 122 mL [17–562] on D-1). On weaning day, 52% of patients were still on norepinephrine (median dose 0.22 μg/kg/min, bitartrate), 72% had a positive cumulative fluid balance (median +5 L), and diuretics were prescribed in 25% of patients. In unadjusted comparisons, patients with successful weaning (63%) had higher urine output at discontinuation and more frequent diuretic use, whereas vasopressor support and fluid balance substantially overlapped between groups. In multivariable analysis, only persistent anuria as the indication for CRRT initiation remained independently associated with weaning failure.

**Conclusions:**

This combined analysis highlights a discrepancy between reported weaning practices and observed bedside management at the centre level. Despite declared reliance on urine output, hemodynamic stability, and decongestion, CRRT is often discontinued under oliguria, residual vasopressor use, and persistent fluid overload. The absence of standardized protocols underscores the need for validated composite readiness criteria that integrate urine output, vasopressor dependency, fluid status, and, possibly, urinary biomarkers.

## Graphical abstract

**Figure:**
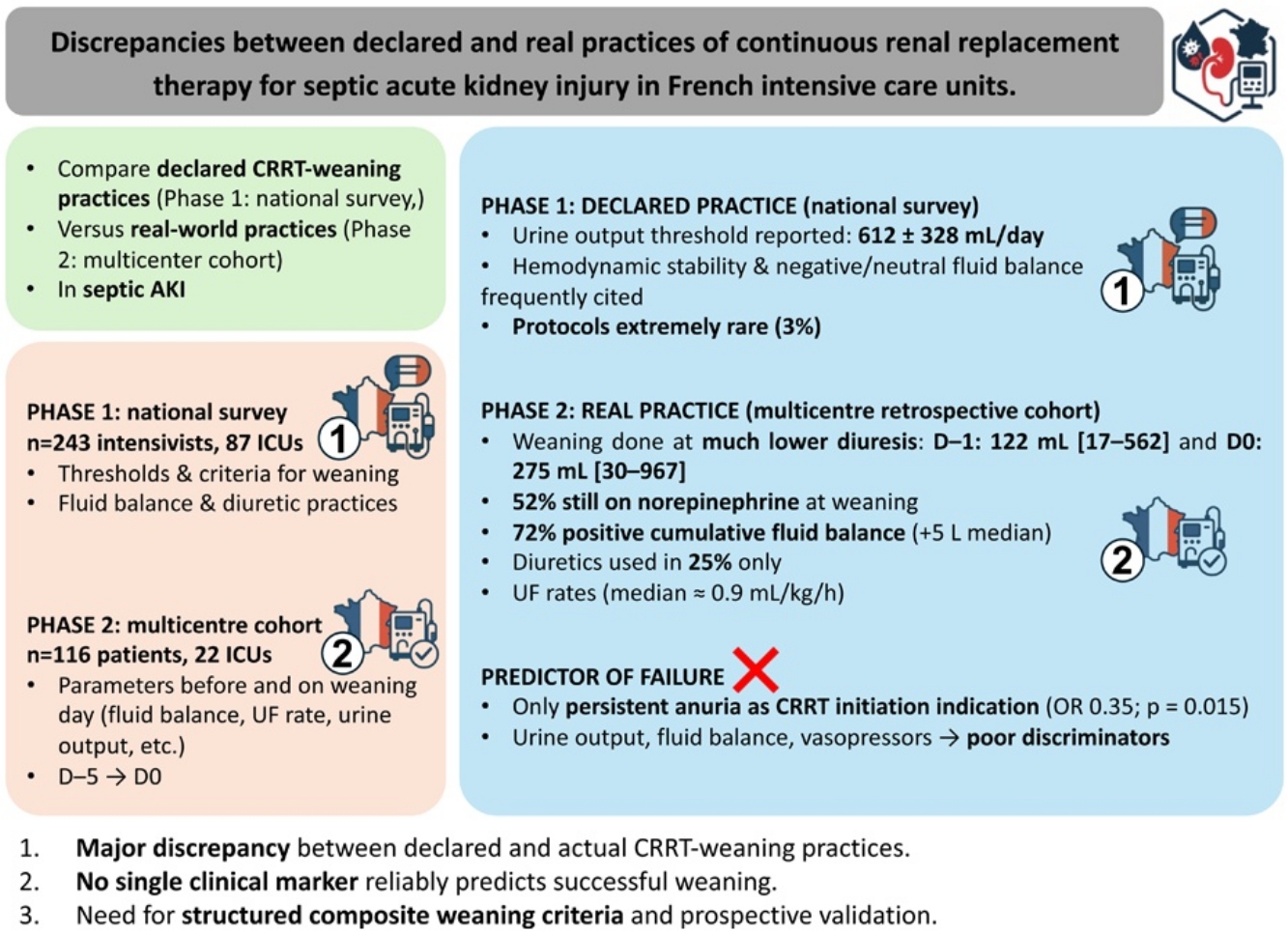

## Background

Acute kidney injury (AKI) is a frequent and severe complication in critically ill patients, affecting more than half of all admissions to intensive care units (ICUs), particularly in the context of sepsis or septic shock [[1], [2], [3]]. Among patients with severe AKI, approximately 10% require renal replacement therapy (RRT), a marker of both the severity of organ dysfunction and a predictor of increased mortality [4,5]. Continuous renal replacement therapy (CRRT) is the most commonly used modality in both French and international ICUs [[6], [7], [8]]. In recent years, multicentre trials have established more precise recommendations for the timing of RRT initiation [[9], [10], [11], [12]]. These studies have clarified indications for starting RRT in critically ill patients with severe AKI, reducing some of the historical uncertainties around early versus delayed initiation strategies.

However, in stark contrast to initiation, the discontinuation of RRT has mainly remained empirical. The 2012 KDIGO guidelines recommend discontinuing RRT when “*it is no longer required due to sufficient renal recovery*” [13]. Yet, they provide no operational definitions or objective thresholds to guide this critical decision. As a result, intensivists must rely on a combination of clinical judgment, hemodynamic evaluation, and laboratory parameters to assess readiness for weaning.

Various clinical and biological markers have been proposed to predict successful RRT discontinuation, including urine output, daily urinary creatinine, daily urinary urea excretion, or kinetic estimated glomerular filtration rate, as well as biomarkers such as cystatin C and neutrophil gelatinase-associated lipocalin (NGAL) [[14], [15], [16], [17], [18], [19]]. Despite promising associations reported in some studies, none of these markers has been universally validated or incorporated into international guidelines [20]. The absence of standard criteria may create substantial variability in practice. Moreover, the consequences of inappropriate timing of weaning can be severe: premature discontinuation may lead to recurrence of metabolic derangements and fluid overload, and the need to restart RRT is consistently associated with worse outcomes, likely reflecting persistent or recurrent severity rather than a direct causal effect [21,22]. Unnecessarily prolonged RRT treatment prolongs exposure to catheter-related complications and may interfere with renal recovery, although this remains difficult to disentangle from underlying illness severity and persistent need for organ support [[23], [24], [25]].

In parallel, recent observational studies have emphasized that the management of RRT cessation is influenced not only by clinical factors but also by organizational and cultural determinants, such as availability of dialysis modalities, staffing expertise, and perceived institutional norms [6,7]. Although surveys and single-center experiences have highlighted these issues, comprehensive multicentre evaluations combining physician-reported practices with actual bedside trajectories are lacking.

Addressing this gap is crucial for informing the development of harmonized protocols and enhancing patient safety and resource utilization in the ICU. To address this gap, we designed a mixed-methods study combining a nationwide survey of declared CRRT weaning practices (Phase 1, survey phase) with a multicentre retrospective cohort describing real-life CRRT discontinuation in patients with septic AKI (Phase 2, retrospective cohort phase). These two components were conceived as complementary approaches to a single research question: the discrepancy between theoretical weaning criteria and bedside decision-making in critically ill patients. By integrating these perspectives, this study seeks to deepen the understanding of current approaches to CRRT discontinuation, identify factors contributing to practice variability, and lay the groundwork for future studies evaluating standardized weaning strategies.

## Methods: study design and data sources

### Study design and setting

We conducted a two-part investigation under a single protocol: (1) Phase 1, a nationwide cross-sectional survey of ICU physicians in France, and (2) Phase 2, a multicentre retrospective cohort of adults with septic AKI undergoing liberation from continuous renal replacement therapy (CRRT).

Importantly, 20 of the 22 centres participating in Phase 2 (multicentre retrospective cohort) also contributed to the Phase 1 (survey), strengthening the integration between declared practices and observed bedside decisions.

To limit clinical heterogeneity, this study was restricted to patients with septic AKI, the most frequent indication for CRRT in the ICU, in whom hemodynamic and fluid management constraints strongly influence weaning decisions.

Both components complied with the Declaration of Helsinki and were approved by the Bordeaux University Hospital Ethics Committee (CER-BDX 2024-142).

The primary objective of Phase 1 (survey phase) was to characterize intensivists’ declared criteria and definitions for CRRT weaning in septic AKI at the national level. The primary objective of Phase 2 (retrospective cohort phase) was to describe real-world trajectories and outcomes of CRRT discontinuation in septic AKI patients and to identify factors associated with successful weaning.

These two components were prospectively conceived as complementary approaches addressing the same overarching objective: comparing declared CRRT weaning practices with real-life bedside decisions within the same healthcare context.

### Phase 1: declared CRRT weaning practices (national survey)

#### Participants and data collection

Between 30 August and 30 October 2023, we disseminated a 45-item electronic questionnaire by email to 230 ICUs across mainland France and overseas territories (pediatric ICUs excluded). Eligible participants were board-certified physicians regularly involved in prescribing and managing CRRT; physicians working in ICUs where CRRT is not available were excluded, whereas centers exclusively using continuous techniques were eligible.

The survey was hosted on SKEZIA, a GDPR-compliant platform. Participation was voluntary, anonymous, and without incentive. The platform restricted submissions to one response per physician account to prevent duplicates. Survey responses were analysed at the physician level. In centres with multiple respondents, divergent answers were not collapsed into a single centre-level response, as the objective was to capture individual practices rather than infer institutional protocols.

The questionnaire captured respondent demographics and training, criteria and thresholds considered for CRRT weaning, organizational and decision-making processes for discontinuation, and perceived consequences of early versus delayed weaning on renal outcomes.

A scenario-based question exploring fluid management strategies under CRRT was included to capture clinicians’ attitudes toward decongestion in different haemodynamic contexts; given its exploratory nature, results are presented in the Supplementary Material.

Reporting adheres to CHERRIES (recruitment, consent/anonymity, duplicate protection, completion metrics, and data handling). The full questionnaire and response/completion rates are provided in the Supplementary Material.

### Phase 2 (multicentre retrospective cohort phase): Observed CRRT weaning practices

Having described and declared CRRT weaning practices, we next examined real-life CRRT discontinuation trajectories in the Phase 2 cohort.

#### Participants

Between September 2024 and March 2025, this multicentre retrospective study was conducted across 22 ICUs in metropolitan France and overseas territories. All centers had access to both continuous and intermittent renal replacement therapy (RRT) modalities. The inclusion criteria were as follows:

- Age ≥18 years,
- ICU admission for sepsis or septic shock, as defined by Sepsis-3 criteria,
- KDIGO stage 3 acute kidney injury requiring initiation of continuous renal replacement therapy (CRRT) as first-line modality,
- The first episode of definitive CRRT discontinuation during the ICU stay, regardless of subsequent re-initiation.

To limit selection bias, consecutive patients within each centre were recruited. Investigators were instructed to retrospectively identify eligible cases starting from the most recent CRRT discontinuation episodes and moving backward chronologically. Each centre was asked to include approximately five consecutive eligible patients; a limited range (2–7 patients) was allowed to accommodate centre-specific feasibility constraints inherent to retrospective data collection and to avoid over-representation of high-volume centres. Patients were identified using routinely available local data sources, including CRRT or dialysis logs, electronic medical record prescriptions, and dialysis machine software, as specified in the study data collection instructions.

Patients were excluded if they received intermittent RRT as the initial treatment, had treatment limitation or withdrawal decisions before any weaning attempt, or died before a discontinuation decision could be made.

#### Data collection

A standardized electronic case report form (CRF) was developed and securely hosted on the Bordeaux University Hospital Nextcloud platform. Trained investigators retrospectively abstracted data from medical records.

#### Definitions

In Phase 2 (retrospective cohort phase), the analyzed CRRT weaning episode was defined as the first attempt to discontinue CRRT.

Successful weaning was defined as the absence of any re-initiation of RRT within 7 days following discontinuation, a definition commonly used in adult CRRT liberation studies to distinguish sustained weaning from transient interruption and to allow comparability across cohorts [[26], [27], [28]].

Baseline serum creatinine was defined as the most recent value available before ICU admission. When no reference value was available, baseline creatinine was estimated using an inverse MDRD equation assuming a reference estimated glomerular filtration rate (eGFR) of 75 mL/min/1.73 m^2^ [29]. Baseline eGFR was then calculated from this creatinine value.

Net ultrafiltration was expressed in mL/kg/h to allow normalization for body weight and to reflect the intensity of fluid removal at the bedside. For reference, 1 mL/kg/h corresponds approximately to 24 mL/kg/day when ultrafiltration is applied continuously.

For all analyses, D0 was defined as the first intentional discontinuation of CRRT performed with the clinical objective of weaning, irrespective of whether intermittent RRT was initiated afterward. The days preceding weaning were labeled relative to D0 (e.g., D-1, D-2), allowing assessment of clinical, hemodynamic, and biochemical parameters in the period immediately preceding discontinuation.

Norepinephrine doses are reported as administered in routine clinical practice (norepinephrine bitartrate), expressed in μg/kg/min.

#### Statistical analysis

Descriptive statistics were used to summarize survey responses and cohort characteristics. Categorical variables are presented as counts (percentages) and were compared using chi-squared or Fisher’s exact tests, as appropriate. Continuous variables are presented as means ± standard deviation or medians with interquartile ranges, depending on distribution, and compared using Student’s t-test or Wilcoxon rank-sum test.

Daily weaning-related parameters are presented descriptively to illustrate temporal trajectories preceding CRRT discontinuation.

Correlation between urine output and net ultrafiltration (UF) volumes during the five days preceding CRRT discontinuation was assessed using linear regression and reported as R^2^ values. No imputation was performed for missing data.

Variables associated with the outcome in univariate analysis (p < 0.10) and those deemed clinically relevant a priori were included in the multivariable logistic regression model. The linearity-in-the-logit assumption for continuous variables was assessed using restricted cubic splines. Potential first-order interactions between SAPS II, urine output on D-1, and anuria as the indication for CRRT initiation were explored and were not retained in the final model. To limit overfitting and collinearity, the number of candidate variables was restricted and redundant variables were excluded (e.g. age, already captured within SAPS II). Only variables available at the time of CRRT initiation or weaning decision with complete data were considered.

Analyses were conducted using Jamovi (version 2.4) and R statistical software (version 4.3.1; R Foundation for Statistical Computing, Vienna, Austria). All tests were two-sided, and p-values <0.05 were considered statistically significant.

### Sensitivity analyses

Several prespecified sensitivity analyses were performed to test the robustness of the main findings.

*Centre-level analysis and comparison of declared practices and observed CRRT discontinuation practices:*

Each patient was linked to an anonymised centre identifier. Centres were classified according to their participation in the Phase 1 (survey) (defined as at least one physician respondent), allowing comparison between declared practices (Phase 1) and observed CRRT discontinuation practices (Phase 2) within common centres. This centre-restricted sensitivity analysis was designed to align observed weaning practices with centres that also contributed declared practices; therefore, centres without Phase 1 participation (Dax and Nantes) were excluded. As a sensitivity analysis of successful CRRT weaning, the primary multivariable model was refitted using a mixed-effects logistic regression with a random intercept for centre to account for potential clustering of practices within centres.

*Sensitivity analysis using a prespecified urine-output threshold:*

In a complementary sensitivity analysis, urine output on D-1 was additionally analysed using a prespecified clinical threshold (<500 vs ≥500 mL/24 h), while reference serum creatinine was retained as a continuous variable (Supplementary Table S5).

*Sensitivity analysis excluding constrained CRRT interruptions:*

Because intention at the time of CRRT cessation may be uncertain in retrospective data, an additional sensitivity analysis was performed after exclusion of CRRT cessations classified as constrained interruptions, identified through structured chart review (technical or organisational reasons, such as early switch to intermittent haemodialysis or repeated circuit thrombosis). The multivariable model was refitted using the same covariates as in the primary analysis.

## Results

### Phase 1 (national survey)

#### Respondent characteristics

A total of 243 intensivists from 87 ICUs across France completed the survey; the overall completion rate was 36%, and no partial response was included in the analysis. Respondents were distributed across metropolitan France and overseas territories and represented ICUs with a median of 13 beds [[10], [11], [12], [13], [14], [15], [16], [17], [18]] (Supplementary Material, Figure S1). The majority worked in general public hospitals (55%, 135/243) or university hospitals (39%, 95/243), and 6% (14/243) in private institutions. Sixty-six percent (161/243) practiced in mixed medical-surgical ICUs, 31 % (75/243) in medical ICUs, and 3% (7/243) in surgical ICUs. Overall, 54% (131/243) of respondents had over 10 years of ICU experience, including 25% (61/243) with more than 20 years of experience, while 18% (44/243) had less than 5 years of experience. Nearly half (46%, 112/243) reported having formal training or a university diploma in RRT, and 12% (30/243) were designated as the lead physician for RRT in their department. Seventy-seven percent reported access to both continuous and intermittent RRT modalities, whereas 23% (57/243) used only CRRT in their ICU. Among those with access to both modalities, CRRT was still preferred in cases of hemodynamic instability (78%). For 149/243 (61%) respondents, CRRT was favored due to the unavailability of intermittent RRT or lack of trained staff. Respondents could select multiple reasons for preferring CRRT; therefore, percentages exceed 100%. All these data are presented in Table 1.

**Table 1:** Characteristics of participating institutions and ICUs.

Characteristics of participating institutions and ICUs (n = 243)	
Characteristics	Data
Age	
<30 years	7 (3%)
30−40 years	105 (43%)
40−50 years	67 (28%)
50−60 years	40 (16%)
>60 years old	24 (10%)
Experience	
<5 years	43 (18%)
5−10 years	68 (28%)
10−20 years	71 (29%)
20−30 years	41 (17%)
>30 years	20 (8%)
Type of hospital	
University hospital	95 (39%)
Public non-university hospital	135 (55%)
Private non-university hospital	14 (6%)
Type of ICU	
Medical	74 (31%)
Surgical	7 (3%)
Mixed	161 (66%)
University diploma or formal training in RRT	
Yes	112 (46%)
No	131 (54%)
Department RRT lead	
Yes	30 (12%)
No	213 (88%)
RRT modalities available	
Continue RRT only	57 (23%)
Continue and Intermittent RRT	186 (77%)
Reason for preferring CRRT*	
Hemodynamic instability	190 (78%)
Availability of the technique in the ICU	100 (41%)
Easier management of drug prescription (including antibiotics)	75 (31%)
Staff trained only for continuous RRT	49 (20%)
Reduced risk of osmotic disequilibrium syndrome	45 (19%)
Easier correction of acid-base disorders	31 (13%)

ICU: intensive care unit; RRT: renal replacement therapy.

Data are expressed as n (%).

* Multiple responses were allowed; percentages therefore do not sum to 100%.

### Declared criteria considered for initiating CRRT weaning

Regarding the weaning strategy, 60/243 (25%) physicians described practicing “early weaning”, while only 3/243 (1%) described a “late weaning” strategy. Most (179/243, 74%) reported an intermediate approach. No predefined cut-off or operational definition was provided in the questionnaire, and these categories therefore reflect physicians’ self-perceived practice. These categories were self-reported and are presented for descriptive purposes only, without implying standardized or comparable clinical strategies.

**Fig. 1:**
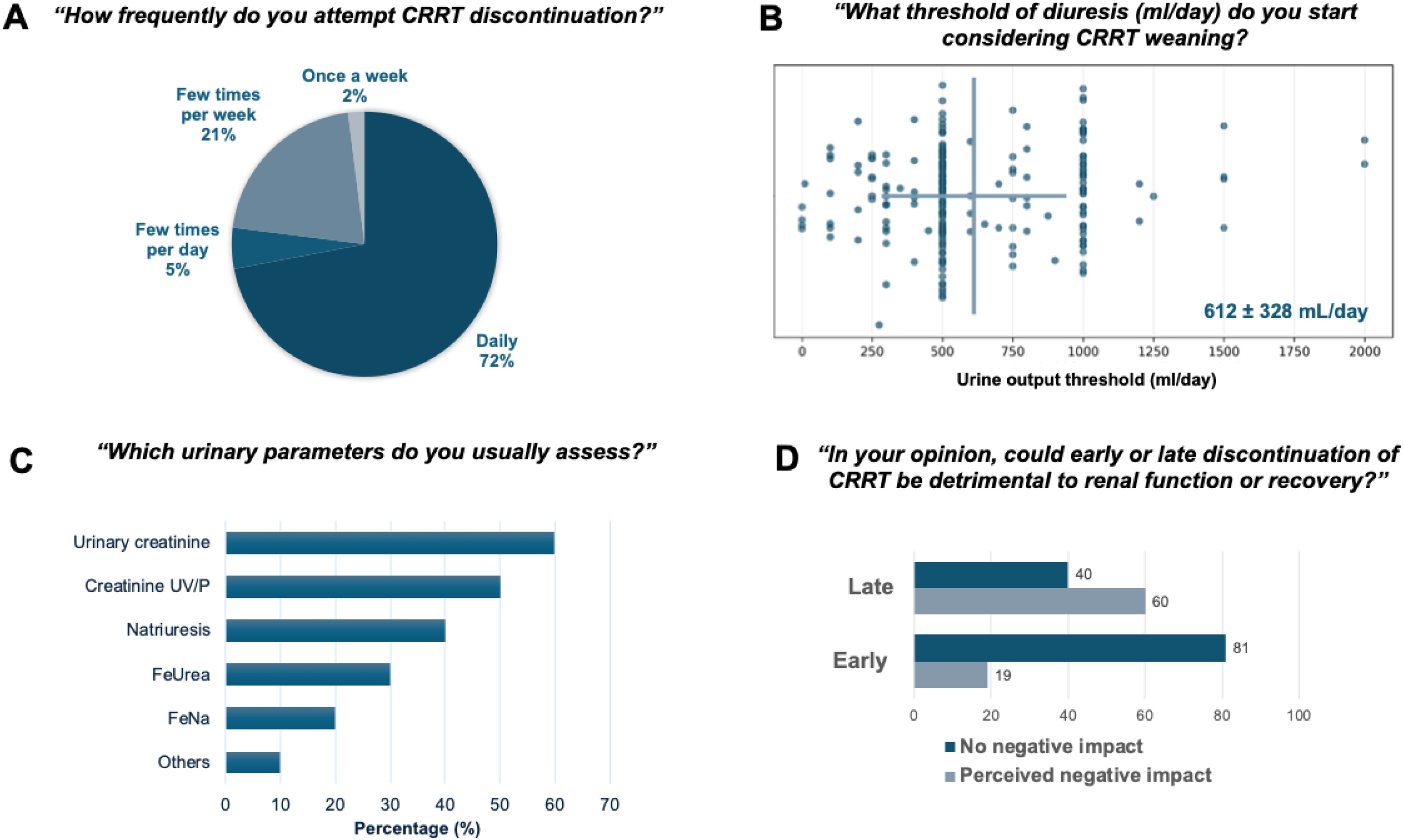
Declared practices and perceptions regarding CRRT weaning in septic AKI (Phase 1, national survey, n = 243). Panel A. Reported frequency of considering CRRT weaning. Panel B. Urine output thresholds considered for continuous renal replacement therapy weaning in patients initially anuric. Panel C. Urinary biochemical analyses performed in patients with preserved diuresis and markers most frequently assessed. Panel D. Perceived risk of early versus late discontinuation of CRRT on renal function or recovery. Categories are self-reported and non-standardized. AKI: acute kidney injury; CRRT: continuous renal replacement therapy; FeNa: fractional excretion of sodium; FeUrea: fractional excretion of urea.

Most physicians (175/243, 72%) reported considering weaning daily (Fig. 1A). The primary trigger for considering CRRT weaning was the return of spontaneous urine output in previously anuric patients (225/243; 93%). The mean urine output threshold declared by clinicians to initiate weaning was 612 ± 328 mL/day (Fig. 1B). Among respondents, 146/243 (60%) reported performing urinary biochemical analyses in patients with preserved residual urine output. Whenever used, biomarkers included urinary urea (26%), urinary creatinine (23%), creatinine clearance (UV/P, 17%), and natriuresis (16%) (Fig. 1C). These analyses were performed daily in 40% of cases and preferentially based on 24 -h urine collections (60%).

Pre-existing chronic kidney disease was not considered a major determinant of weaning strategy, as 154/243 respondents (63%) reported it did not influence their practice.

While 205 out of 243 physicians (81%) claimed that the cost of RRT consumables did not influence weaning decisions, 117/243 (46%) did not interrupt CRRT until the filter lifespan was completed, even if clinical criteria suggested weaning was appropriate.

### Decision-making modalities for CRRT discontinuation

Fluid status was a key reason for continuing CRRT, with 116 out of 243 (48%) respondents stating they “often” or “sometimes” used CRRT solely for fluid management. Detailed results of the scenario-based questions on fluid management are provided in the Supplementary Material (Figure S5). Strikingly, 42% (102/243) stated they never prescribed diuretics during ongoing CRRT. When net UF caused hemodynamic instability, 75% (180/243) preferred reducing the net UF rate, while 25% (63/243) escalated vasopressor support.

Although 72% (175/243) of respondents reported assessing weaning readiness daily, only 3% (3/243) had a formal written protocol.

### Perceptions of risks and definitions of successful weaning

Sixty percent of respondents considered delayed discontinuation potentially detrimental to renal recovery, whereas 19% (46/243) perceived early weaning as potentially harmful due to risks of metabolic instability and RRT re-initiation (Fig. 1D). Definitions of successful weaning were heterogeneous: 34% (82/243) considered success to be the absence of RRT resumption within 7 days, 24% (58/243) at ICU discharge, and 15% (36/243) at hospital discharge. Other responses, including alternative definitions, were less frequently reported. Sixty-nine percent reported transitioning to intermittent RRT before final discontinuation. Timing of dialysis catheter removal also varied: 94 out of 243 (39%) intensivists removed it after 72 h when weaning was deemed definitive, 68/243 (28%) after 48 h, and 51/243 (21%) after 24 h.

### Phase 2 (retrospective cohort phase)

#### Patients’ characteristics

To contextualize these declared practices, a multicentre retrospective cohort of 116 patients in 22 centres with septic AKI requiring CRRT was analyzed (Supplementary Material, Figure S2). The median age was 65 years [56−72], and 68% (79/116) of the participants were male. The median SAPS II score at admission was 70 [54–82]. The median BMI was 28.1 [24.3–31.5] and the median baseline estimated glomerular filtration rate (GFR) was 72.6 [45.2–91.1] mL/min/1.73 m^2^.

The main indication for initiating CRRT was persistent anuria (34%, 40/116), followed by medically resistant acidosis (33%, 38/116) (Table 1). The median time from ICU admission to KDIGO stage 3 AKI onset was 0 [0–1] day, and from ICU admission to CRRT initiation was 1 [1–4] day. The median time between CRRT initiation and the first disconnection was 3 [1–4] days. Of the 116 patients, 73 (63%) were definitively weaned after the first CRRT disconnection. The median duration before definitive weaning was 4 [2–7] days. Considering all RRT modalities, the total median duration of RRT was 7.5 [3–14] days. All these data are presented in Table 2.

**Table 2:** Description of the study population.

Description of the study population	n = 116
*Baseline characteristics*	
Male*, n (%)*	79 (68%)
Age (years)*, median [IQR]*	65 [56−72]
BMI*, median [IQR]*	28.1 [24.3–31.5]
SAPS II*, median [IQR]*	70 [55–82]
SOFA*, mean ± SD*	12 ± 4
Baseline serum creatinine (μmol/L)*, median [IQR]*	85 [71–129]
Baseline eGFR (mL/min/1.73 m^2^)*, median [IQR]*	72.6 [45.2–91.1]
Delay between ICU admission and AKI, *median [IQR]*	0 [0–1]
Delay between ICU admission and CRRT initiation*, median [IQR]*	1 [1–3]
*Indication for CRRT initiation, n (%)*	
Anuria	40 (34.4%)
Acidosis	38 (32.7%)
Hyperkalemia	19 (16.3%)
Pulmonary edema	8 (6.9%)
Other	7 (6%)
Uremia > 40 mmol/L	4 (3.4%)
*Clinical outcomes*	
Length of hospital stay (days)*, median [IQR]*	20 [11–29]
Death*, n (%)*	22 (19%)
Delay between initiation of CRRT and first CRRT weaning (days), *median [IQR]*	3 [1–4]
Total duration of CRRT, from initial to final discontinuation (days), *median [IQR]*	4 [2–7]
Total duration of CRRT, from initiation to technique weaning (days), *median [IQR]*	7.5 [3–14]

Detailed characterization of patient demographics, clinical indications for continuous renal replacement therapy, and the duration of extracorporeal renal support.

Baseline demographic characteristics and severity scores were collected at ICU admission. Baseline serum creatinine was defined as the most recent value available before ICU admission; when unavailable, it was estimated using an inverse MDRD equation assuming a reference eGFR of 75 mL/min/1.73 m². Baseline eGFR was calculated from this creatinine value. Baseline serum creatinine was directly available in 101/116 patients (87%) and estimated using the inverse MDRD equation in 15/116 patients (13%).

Indications for CRRT initiation and delays are reported at the time of CRRT initiation. Outcomes were assessed during the ICU stay.

“Other indications” corresponded to CRRT initiation in the context of severe multi-organ failure or clinician-driven decision making, when classical predefined thresholds for RRT initiation (e.g. AKIKI criteria) were not met, but CRRT was deemed necessary based on overall clinical severity.

AKI: acute kidney injury; BMI: Body Mass Index; CRRT: Continuous Renal Replacement Therapy; eGFR: estimated Glomerular Filtration Rate; ICU: Intensive Care Unit; SAPS: Simplified Acute Physiology Score; SD: standard deviation; SOFA: Sequential Organ Failure.

#### Clinical and fluid status during the pre-weaning phase and on weaning day (D0)

**Fig. 2:**
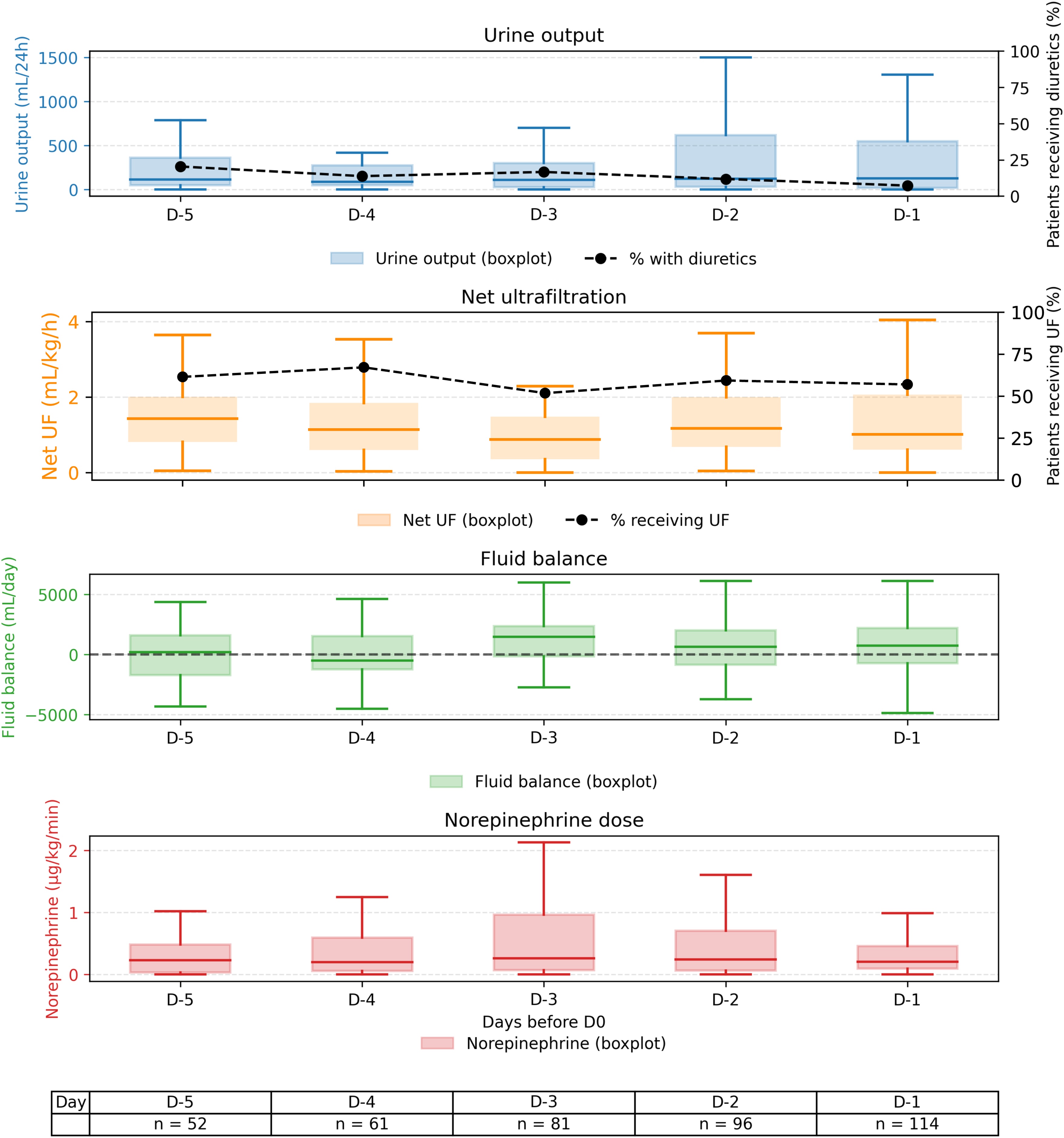
Description of weaning-related parameters from day −5 to day 0 preceding the final discontinuation of continuous renal replacement therapy. Daily evolution of urine output, net ultrafiltration, diuretic use, and vasopressor support in the five days preceding CRRT discontinuation (D-5 to D0), stratified by weaning outcome. Boxplots represent median and interquartile range; whiskers indicate the 1.5× interquartile range. Dashed lines indicate the proportion of patients receiving diuretics or vasopressors at each time point. The number of patients on each day is shown below the panels.

In the five days preceding weaning (D-5 to D-1), the median daily urine output remained stable, peaking at 122 [17–562] mL on D-1. Diuretic use decreased progressively from 20% (10/50 patients) on D-5 to 7% (8/113 patients) on D-1. Net UF was applied variably: from 71% (42/59) on D-4 to 58% (65/113) on D-1. The median daily net UF volume, normalized by the number of net UF days preceding the weaning period, was 1767 [1015–2946] mL (approximately 74 mL/h or 0.92 mL/kg/h). The cumulative fluid balance (FB) was positive on all days preceding weaning except on D-4 (−486 [−1270 to 1550] mL). The normalized cumulative FB median across the CRRT period was +1049 [−16 to 2188] mL. All of these data are detailed in Fig. 2.

**Fig. 3:**
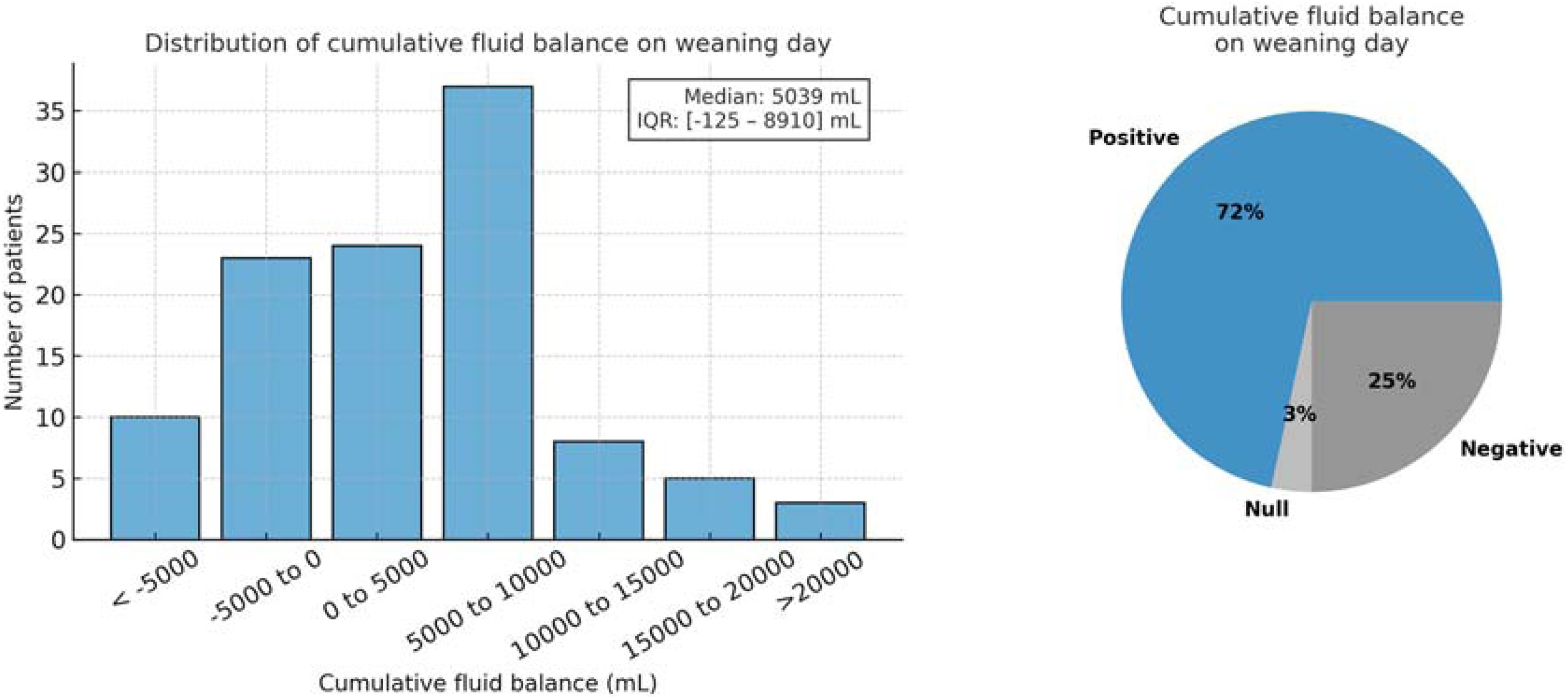
Cumulative fluid balance on the day of definitive weaning from continuous renal replacement therapy.

On the day of weaning (D0), half of the patients (60/116, 52%) were still on norepinephrine with a median dose of 0.22 [0.10−0.60] μg/kg/min (bitartrate). The cumulative FB on D0 was positive in 72% (83/116) of patients, negative in 25% (29/116), and neutral in 3% (4/116), with a median of +5039 [−125 to +8910] mL (Fig. 3). The median urine output on D0 was 275 [30–967] mL (Supplementary Figure S3), and 25% (29/116) of patients received diuretics.

Urine output and net UF volumes were only weakly and inconsistently correlated in the days preceding weaning (Supplementary Material, Figure S4).

#### Comparison between successful and failed weaning

Among all patients, 73 (63%) experienced successful first-attempt weaning, defined as no resumption of CRRT within 7 days. Baseline characteristics were broadly similar between groups, although patients with successful weaning had higher SAPS II scores (73 vs. 65; p = 0.045) (Supplementary Material, Table S1). Persistent anuria as the RRT initiation trigger was more frequent in patients with weaning failure (51% vs. 25%), whereas metabolic acidosis was more common in the success group (38% vs. 23%; overall p = 0.011). ICU length of stay was longer in the failure group (26 vs. 19 days; p = 0.008), and ICU mortality was higher (33% vs. 11%; p = 0.009).

**Fig. 4:**
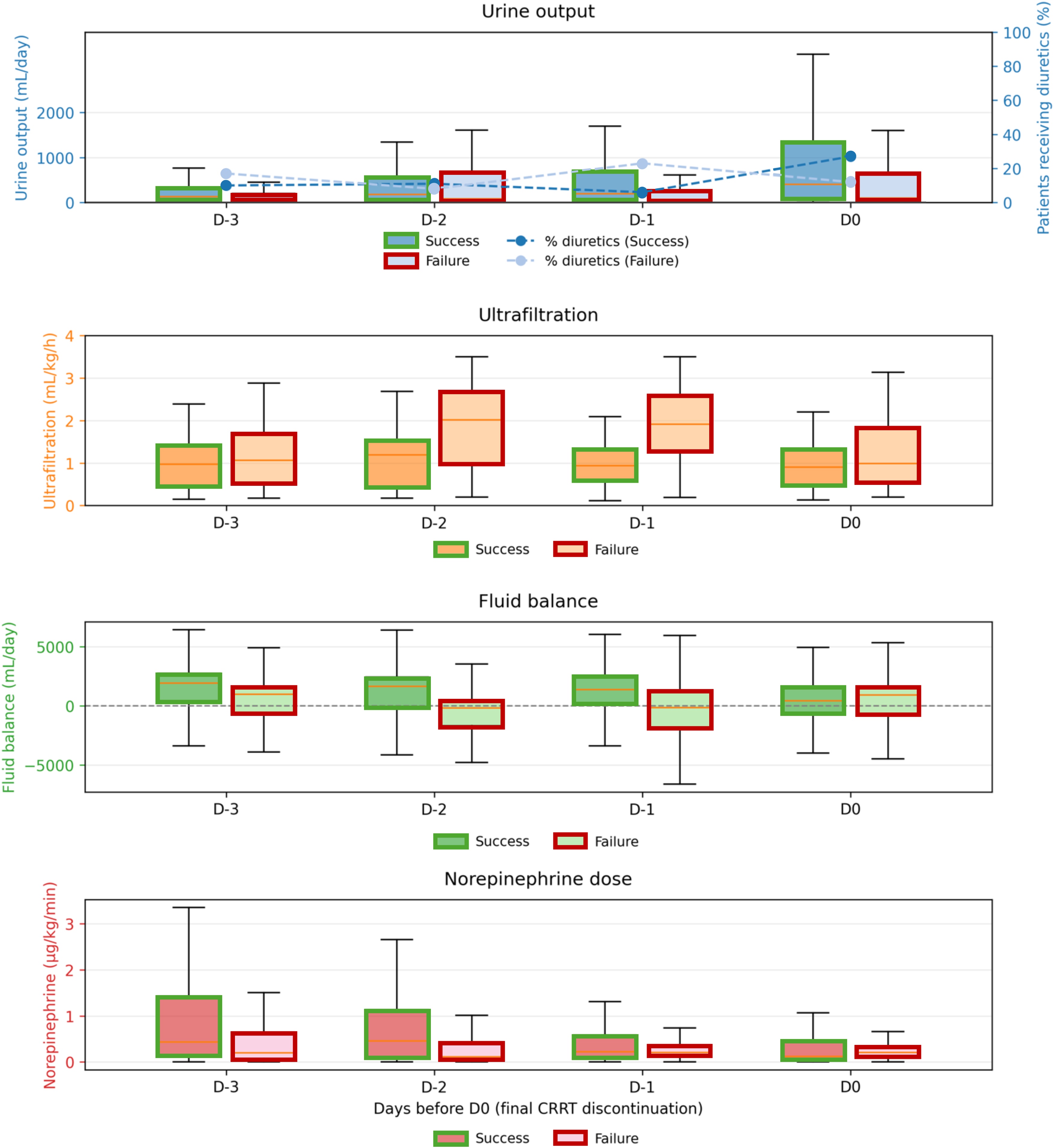
Comparison of weaning parameters from day −3 to day 0 before final CRRT discontinuation between successful and failure weaning groups. Daily trajectories of urine output, net ultrafiltration rate, cumulative fluid balance, and norepinephrine dose (bitartrate) from day −3 to day 0 (final CRRT discontinuation), stratified according to first weaning outcome (successful vs. failure). Data are presented as boxplots (median, interquartile range, and whiskers). The outline color indicates the weaning outcome (green: success, red: failure). The proportion of patients receiving diuretics or net ultrafiltration is shown as dashed lines on a secondary axis where applicable. These data are presented for descriptive purposes only to illustrate clinical patterns preceding CRRT discontinuation and were not used for confirmatory statistical inference.

In the three days preceding CRRT discontinuation, urine output tended to increase over time in patients with successful first weaning, whereas it remained low and relatively stable in patients who subsequently required RRT resumption. On the day of discontinuation, median urine output was higher in the successful group (400 mL [54–1350]) than in the failure group (100 mL [18–650]). Diuretic use appeared more at the time of discontinuation among successfully weaned patients, while net ultrafiltration volumes per kilogram were generally higher in patients who ultimately failed weaning, particularly in the days preceding discontinuation. Cumulative fluid balance remained positive in both groups, with substantial overlap throughout the observation period. These comparisons are presented for descriptive purposes only. These trajectories are presented descriptively to illustrate clinical patterns preceding CRRT discontinuation and should not be interpreted as evidence of causal or independent associations (Fig. 4; Supplementary Material Table S2).

In multivariate analysis, only persistent anuria as the indication for CRRT initiation remained independently associated with weaning failure (OR 0.35, 95% CI 0.15−0.81; p = 0.015) (Table 3). Neither SAPS II, urine output on day −1, nor baseline serum creatinine were significantly associated with outcomes. No meaningful departure from log-linearity was identified for SAPS II, urine output on D-1, or reference serum creatinine (p for non-linearity: 0.50, 0.39, and 0.44, respectively). These variables were therefore retained as continuous linear terms in the final model.

**Table 3:** Univariate and multivariate analyses of factors associated with successful CRRT weaning.

	Univariate analysis			Multivariate analysis		
Variable	OR	CI95%	p-value	OR	CI95%	p-value
SAPS II	1.019	1.0−1.039	0.051	1.009	0.991−1.027	0.316
Urine output on D-1 (per 100 mL)	1.053	0.976−1.135	0.181	1.044	0.969−1.124	0.256
Anuria (indication for CRRT initiation)	0.364	0.155−0.855	0.020	**0.346**	**0.147−0.813**	**0.015**
Reference serum creatinine (per μmol/L)	0.997	0.992−1.003	0.406	0.996	0.990−1.002	0.169

Variables with p < 0.10 in univariate analysis or deemed clinically relevant were entered into the logistic regression model. Collinear variables (e.g., age, already included in SAPS II) were excluded. Model performance was acceptable (AIC = 145.6; BIC = 159.2), with no evidence of multicollinearity (all VIF < 1.1).

#### Sensitivity analyses

Among the 22 centres contributing CRRT discontinuation episodes, 20 centres had also participated in the Phase 1 (national survey), enabling comparison between declared and observed practices within the same institutions (Supplementary Table S3).

In a sensitivity analysis accounting for centre-to-centre variation, the main results were unchanged. In practical terms, this suggests that the association between anuria at CRRT initiation and unsuccessful weaning was not driven by a small number of centres with specific local practices (Supplementary Table S4).

When urine output on D-1 was analysed using a prespecified threshold (<500 vs ≥500 mL/24 h), the multivariable associations remained consistent with the primary model, including the independent association of anuria as the initial CRRT indication with weaning failure (Supplementary Table S5).

Excluding CRRT cessations classified as constrained interruptions did not alter the main results (Supplementary Table S6).

A summary table contrasting declared and observed practices is provided in the Supplementary Material (Supplementary Table S7).

## Discussion

The originality of this work lies in the integrated comparison of declared and observed CRRT weaning practices, using a mixed-methods approach to address a single clinical question from complementary perspectives.

This combined analysis of a nationwide survey (Phase 1) and a multicentre retrospective cohort (Phase 2) provides a unique perspective on CRRT weaning practices in critically ill patients with septic AKI. CRRT weaning in septic AKI remains highly variable, as patients are often discontinued at lower urine outputs, under vasopressors, and with persistent fluid overload despite opposite declared criteria.

A key finding is the marked variability across all stages of the weaning process. In Phase 1 (survey phase), intensivists reported urine output as the main criterion, with a mean threshold of ∼600 mL/day. Yet, Phase 2 (retrospective cohort phase) showed patients were often weaned at much lower values, with a median of only 122 mL [17–562] on the day before discontinuation, below both published thresholds (191−1720 mL/24 h) and the values declared in the survey phase [20,21,30]. Recent French recommendations confirm urine output as the primary parameter to consider, underscoring the gap between perceived best practice and real-life decisions [31]. Taken together, these findings indicate that urine output thresholds are not applied as fixed cut-offs in clinical reality: rather than rigid numbers, clinicians appear to integrate trends (return of urine after anuria), competing priorities (hemodynamic stability under vasopressors), and logistical constraints (filter life, staffing, availability of intermittent RRT). These findings also suggest that clinicians do not rely on a single operational urine-output cut-off, but rather apply heterogeneous and context-dependent decision thresholds. In an exploratory analysis, a steeper increase in urine output in the days preceding CRRT discontinuation was associated with successful weaning (OR 1.33 per 100-mL/day increase in slope, 95% CI 1.12–1.57; p = 0.001). These findings suggest that dynamic changes in urine output may provide additional prognostic information and warrant further investigation. Urine output at weaning nevertheless remained predominantly low in most patients, with only a limited number of higher values.

Some descriptive associations appeared counterintuitive at first glance. In particular, the higher SAPS II observed in successfully weaned patients in univariate analyses likely reflects score composition rather than greater physiological severity and did not persist after multivariable adjustment. Similarly, differences in fluid balance should be interpreted cautiously, as they may reflect clinician-driven strategies rather than physiological readiness for CRRT discontinuation. An apparent contrast emerged between metabolic acidosis and anuria as indications for CRRT initiation. Metabolic acidosis may reflect a more reversible condition, whereas persistent anuria likely reflects more severe renal dysfunction, consistent with its association with weaning failure in the present study.

Hemodynamic status also diverged from declared practices. Although survey respondents emphasized stability without vasopressors, in Phase 2 (retrospective cohort phase), nearly half of patients were weaned under norepinephrine at low-to-moderate doses, suggesting that residual support is often accepted in practice. At the time of CRRT discontinuation, a substantial proportion of patients remained on norepinephrine, with marked inter-individual variability in vasopressor dose. Observational studies report conflicting associations between vasopressor use and successful liberation, underscoring that no single hemodynamic parameter can reliably guide this decision [32].

Fluid management illustrates the persistent gap between declared and observed practices. In Phase 1 (survey phase), many intensivists reported maintaining CRRT solely to achieve a neutral or negative balance. Yet, Phase 2 (retrospective cohort phase) showed that 70% of patients still had a positive cumulative balance on D0 (median +5 L), with net UF applied in fewer than 60% of cases and diuretics prescribed to only one in five patients. This discrepancy contrasts with the literature linking fluid overload to increased mortality, but it likely reflects a strategy constrained by hemodynamic instability rather than deliberate tolerance of fluid accumulation. Still, the modest net UF rates (median 0.92 mL/kg/h) are consistent with prior reports in hemodynamically unstable patients [33] and fall below the range (1.0–1.75 mL/kg/h) that observational studies have associated with the best outcomes [[34], [35], [36]]. Such patterns suggest that competing risks, particularly hypotension and concern for renal ischemia, drive a cautious approach. Embedding structured fluid targets (e.g., neutral to mildly negative balance when feasible) and encouraging earlier diuretic use once urine output resumes could help reconcile intentions with practice while minimizing hemodynamic harm [37,38].

The heterogeneity in defining successful weaning further illustrates the lack of standardization. In Phase 1 (survey phase), only a third of respondents considered seven days without RRT resumption sufficient, and nearly one in five equated success with ICU discharge. This mirrors the absence of validated criteria in current guidelines, as KDIGO recommendations merely state that RRT may be discontinued once it is “*no longer required*,” leaving practice to local norms and individual judgment [13,31].

Taken together, our findings highlight that CRRT weaning remains a largely unstandardized and clinician-dependent process, reflecting less inadequate care than the absence of evidence-based protocols and the complexity of critically ill patients. These observations are consistent with the conclusions of a recent international Delphi consensus, which likewise emphasized the lack of validated criteria for CRRT liberation and the continued reliance on clinician judgment [32]. In Phase 2 (retrospective cohort phase), higher urine output and diuretic use were associated with successful weaning. Yet, no single parameter, including urine output, fluid balance, or vasopressor use, proved to be a reliable predictor. This echoes prior studies and underscores the need for composite readiness criteria rather than rigid thresholds [20].

Unnecessarily prolonged CRRT may prolong exposure to catheter-related complications and may interfere with renal recovery, although this remains difficult to disentangle from underlying illness severity and persistent need for organ support [39,40]. This underscores the need for standardized decision tools [41], as illustrated by the benefits of structured protocols in mechanical ventilation [42].

Our study has several strengths. To our knowledge, this is the first study to integrate declarative and observational data to systematically assess both perceptions and real-world management of CRRT discontinuation in France. The large sample of clinicians in Phase 1 (survey phase) and the multicentre design of Phase 2 (retrospective cohort phase) increase the generalizability of findings.

Nevertheless, several limitations must be acknowledged. First, given the retrospective observational design, CRRT discontinuation does not always reflect renal recovery alone and may occur for organisational or technical reasons, leading to potential misclassification despite the adjudication of constrained interruptions. Accordingly, our findings pertain to first CRRT discontinuation attempts performed with the intention to stop renal support, in centres with access to alternative RRT modalities, and should be interpreted within this clinical context. Second, despite including 20 centres with consecutive patient inclusion, exhaustive case capture could not be formally verified in the absence of centre-level screening logs; moreover, the predefined target of five patients per ICU (acceptable range 2–7) and unmeasured centre-level factors (such as local staffing or institutional culture) may have introduced residual selection bias. Overall, the results should be interpreted cautiously given the limited sample size. A limitation of Phase 2 (retrospective cohort phase) is the absence of systematic urinary biomarker collection, which may have refined readiness assessment as suggested by recent studies on urinary indices such as urea [43,44]. Finally, although centre-level heterogeneity was addressed using mixed-effects modelling and centre-restricted analyses, residual unmeasured institutional factors cannot be entirely excluded. Haemodynamic characterization remained limited in this retrospective multicentre cohort. A substantial proportion of patients were still receiving vasopressor support at CRRT discontinuation, but detailed features such as shock phenotype or cardiac dysfunction were not available in a standardized manner.

Recent trials such as LIBERATE-D underline the importance of structured RRT liberation strategies, while dedicated studies focusing on CRRT discontinuation are currently underway [40]. Future research should focus on prospective multicentre trials evaluating protocolized weaning strategies and incorporating dynamic markers such as urine output kinetics, urinary biomarkers (e.g., daily urinary urea excretion and urinary creatinine excretion), and hemodynamic parameters (NCT06214390). Establishing clear, reproducible thresholds and decision algorithms could reduce practice heterogeneity and improve patient-centered outcomes, including renal recovery and ICU length of stay.

## Conclusion

In conclusion, CRRT weaning in septic AKI remains highly variable. This study highlights discrepancies between reported practices and observed bedside management at the centre level. While urine output and hemodynamic stability are broadly recognized, real-world decisions often diverge due to patient complexity. These findings underscore the need for prospective studies to define objective criteria and test structured weaning protocols.

## Key-Messages

Reported practices and observed bedside management differ substantially at the centre level: while most intensivists reported requiring urine output >600 mL/day and hemodynamic stability for CRRT discontinuation, in practice many patients were weaned at lower urine outputs, with persistent vasopressor support and positive fluid balances.

Clinical predictors of weaning success are limited: in multivariable analysis, only persistent anuria as the initial indication for CRRT independently predicted weaning failure, whereas urine output, SAPS II, or baseline creatinine did not retain significance.

Future weaning strategies should move beyond single criteria: discrepancies between reported practices and observed bedside management at the centre level underscore the need for structured, composite readiness assessments (urine output, hemodynamics, fluid balance), to be prospectively tested in interventional trials.

## Authors’ contributions

L.S., R.P., C.S., A.B., and A.O. conceived the manuscript. L.S. and A.O. performed the statistical analyses and drafted the manuscript. All authors contributed to data collection, revised the text, approved the final version, and agree to be accountable for its content.

## Consent for publication

Not applicable.

## Ethics approval and consent to participate

This study was approved by the institutional ethics committee of the University Hospital of Bordeaux (approval number: CER-BDX 2024-142). Given its retrospective observational design, the requirement for informed consent was waived in accordance with French regulations.

## Funding

No specific funding was received for this work.

## Competing interests

The authors declare that they have no competing interests.

### Availability of data and materials

The datasets generated and/or analyzed during the current study are available from the corresponding author on reasonable request.
