# Supplementary material for: Discrepancies between declared and real practices of continuous renal replacement therapy for septic acute kidney injury in French intensive care units
Source: Ann Intensive Care. 2026 May 14;16:100077. doi: 10.1016/j.aicoj.2026.100077 (PMC13218118; doi:10.1016/j.aicoj.2026.100077)
Supplement: Supplementary file 1 [file mmc1.docx]

**SUPPLEMENTARY MATERIAL**

**The Supplementary Material provides additional methodological details and secondary or exploratory analyses to support the main findings**

**Figure S1: Geographical distribution of the respondents to the national survey (Phase 1).**

**Figure S2. Geographical distribution of patients enrolled in the Phase 2 (multicentre retrospective cohort) across the French territory.**

**Figure S3. Distribution of urine output at CRRT weaning**


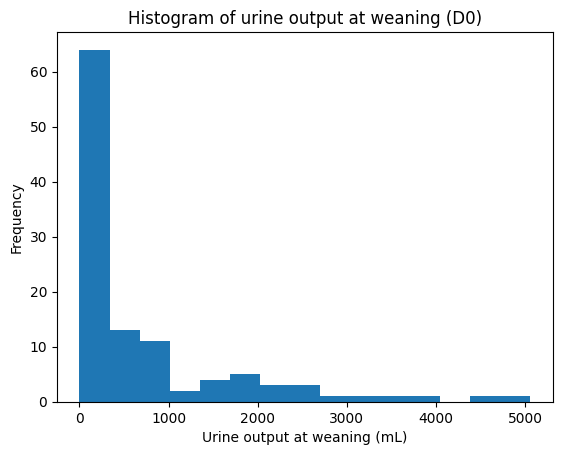


**Figure S4: Correlation between urine output and net** **ultrafiltration (Days -5 to 0)**

**
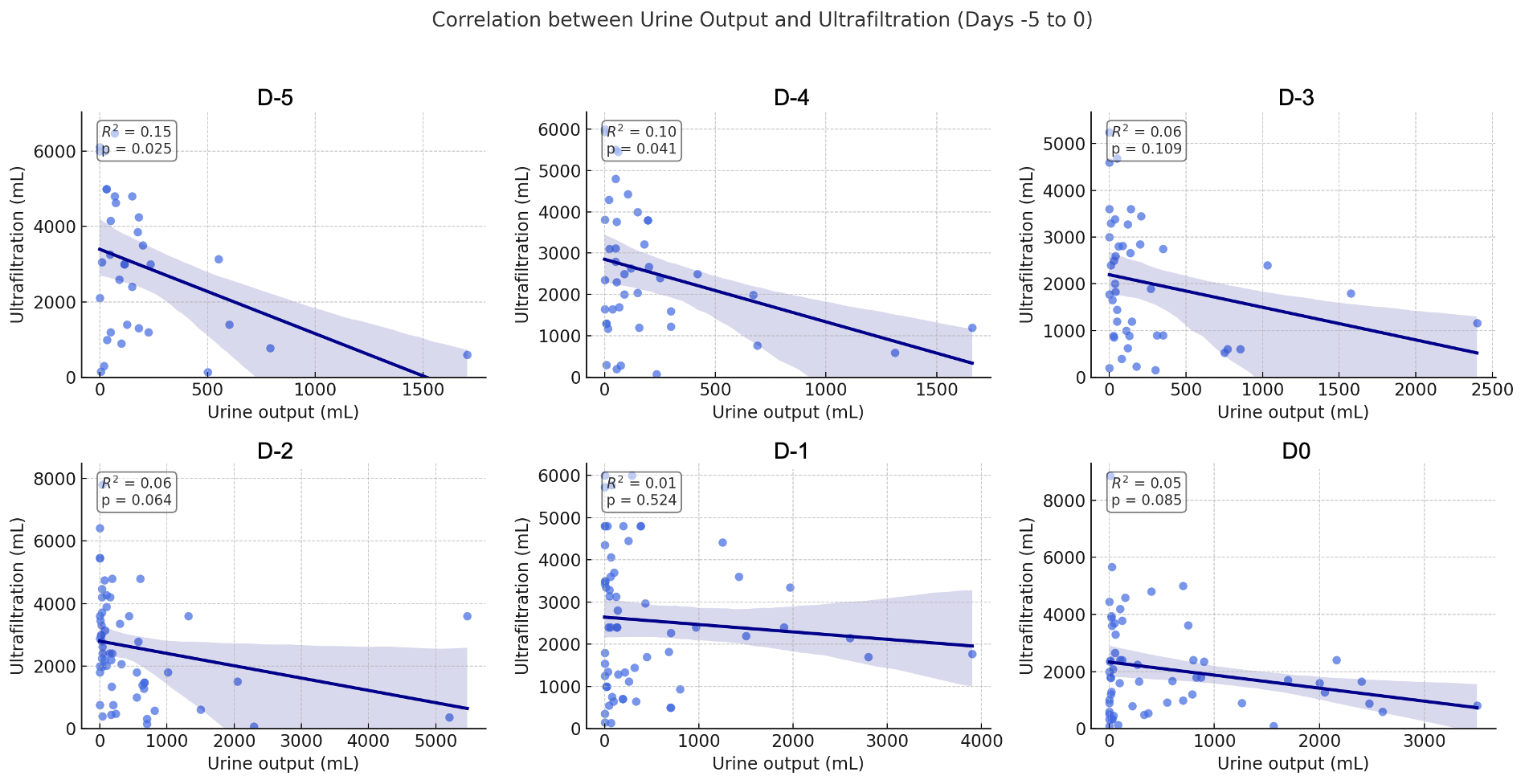
**

**Figure S5. Scenario-based questions on fluid management during CRRT**

To assess attitudes toward fluid management, a short clinical vignette was included in the questionnaire, exploring strategies for managing fluid overload according to the presence or absence of spontaneous urine output and vasopressor support. This scenario aimed to evaluate decision-making consistency across different hemodynamic contexts.

**Table S1: Baseline characteristics and clinical outcomes of patients according to CRRT weaning success or failure (Phase 2: multicentre retrospective cohort).**

| **Variable** | **Successful weaning**  **(n=73)** | **Weaning failure (n=43)** | **p-value** |
| --- | --- | --- | --- |
| **Male sex***, n (%)* | 49/73 (67 %) | 30/43 (70 %) | 0.930 |
| **Age (years)***, median [IQR]* | 67.0 [57.0-73.0] | 62.0 [51.5-70.0] | 0.124 |
| **BMI***, median [IQR]* | 28.4 [23.6-31.4] | 27.9 [24.6-31.7] | 0.938 |
| **SAPS II***, median [IQR]* | 73.0 [58.5-86.0] | 65.0 [48.5-76.0] | **0.045** |
| **SOFA***, median [IQR]* | 12.0 [10.0-14.0] | 12.0 [7.5-14.0] | 0.870 |
| **Baseline serum creatinine (µmol/L)**, *median [IQR]* | 84.5 [71.1-113.0] | 90.0 [72.0-151.0] | 0.412 |
| **Indication for CRRT***, n (%)* |  |  | **0.011** |
| *Treatment-refractory metabolic acidosis* | 28/73 (38%) | 10/43 (23%) |  |
| *Persistent anuria* | 18/73 (25%) | 22/43 (51%) |  |
| *Treatment-refractory hyperkalemia* | 14/73 (19%) | 5/43 (12%) |  |
| *Treatment-refractory pulmonary edema* | 3/73 (4%) | 5/43 (12%) |  |
| *Other* | 7/73 (10%) | 0/43 (0%) |  |
| *Uremia > 40 mmol/L* | 3/73 (4%) | 1/43 (2%) |  |
| **ICU length of stay (days)***, median [IQR]* | 19.0 [10.0-28.0] | 26.0 [16.2-36.2] | **0.008** |
| **ICU death***, n (%)* | 8/73 (11.0%) | 14/43 (32.6%) | **0.009** |

***Baseline characteristics and severity scores were collected at ICU admission. Indications for CRRT reflect the primary reason for CRRT initiation. Clinical outcomes were assessed during the ICU stay***

***BMI: body mass index; CRRT: continuous renal replacement therapy; ICU: intensive care unit; IQR: interquartile range; SAPS II: Simplified Acute Physiology Score II; SOFA: Sequential Organ Failure Assessment.***

**Table S2: Comparison of weaning parameters from day -3 to day 0 before final CRRT discontinuation between successful weaning and weaning failure groups.**

|  | **Group** | **D-3** | **D-2** | **D-1** | **D0** |
| --- | --- | --- | --- | --- | --- |
| **Sample size** | **First weaning success: Success** | 42 | 56 | 71 | 73 |
|  | **First weaning success: Failure** | 18 | 26 | 40 | 43 |
| **Urine output**  *(ml/day)* | **First weaning success: Success** | 130 [21-318] | 175 [30-556] | 192 [33-700] | 400 [54-1350] |
|  | **First weaning success: Failure** | 70 [30-200] | 80 [22-658] | 65 [8-249] | 100 [18-650] |
|  | **p-value** | 0.664 | 0.510 | 0.059 | **0.011** |
| **Diuretics**  *(yes)* | **First weaning success: Success** | 4/42 (10%) | 6/56 (11%) | 4/71 (6%) | 20/73 (27%) |
|  | **First weaning success: Failure** | 3/18 (17%) | 2/26 (8%) | 9/40 (23%) | 5/43 (12%) |
|  | **p-value** | 0.419 | 1.000 | **0.013** | 0.078 |
| **Net ultrafiltration**  *(ml/kg/h)* | **First weaning success: Success** | 0.97 [0.45-1.42] | 1.19 [0.42-1.55] | 0.94 [0.57-1.33] | 0.90 [0.46-1.33] |
|  | **First weaning success: Failure** | 1.06 [0.54-1.71] | 2.01 [0.98-2.65] | 1.91 [1.28-2.57] | 0.99 [0.53-1.83] |
|  | **p-value** | 0.420 | **0.001** | **<0.001** | 0.150 |
| **Fluid balance**  *(ml/day)* | **First weaning success: Success** | 1912 [300-2759] | 1650 [-182-2454] | 1358 [145-2500] | 413 [-652-1585] |
|  | **First weaning success: Failure** | 968 [-600-1605] | -167 [-1662-422] | -155 [-1903-1242] | 914 [-796-1659] |
|  | **p-value** | **0.023** | **<0.001** | **<0.001** | 0.729 |
| **Norepinephrine**  **(µg/kg/min, bitartrate)** | **First weaning success: Success** | 0.437 [0.149-1.432] | 0.457 [0.108-1.128] | 0.225 [0.096-0.583] | 0.119 [0.054-0.461] |
|  | **First weaning success: Failure** | 0.198 [0.071-0.645] | 0.116 [0.054-0.438] | 0.21 [0.13-0.375] | 0.209 [0.127-0.34] |
|  | **p-value** | 0.291 | 0.079 | 0.891 | 0.590 |

*Weaning-related parameters (urine output, diuretic use, net ultrafiltration, fluid balance, and norepinephrine dose) were collected daily and are presented relative to the day of final CRRT discontinuation (D0). P-values are provided for descriptive purposes only and were not adjusted for multiple comparisons.*

**Table S3. Centre-level overlap between Phase 1 (national survey: declared) and Phase 2 (multicentre retrospective cohort: observed) practices**

| **Variable** | **Value** |
| --- | --- |
| Total patients included in Phase 2 | 116 |
| Total centres contributing to Phase 2 | 22 |
| Centres with ≥1 Phase 1 respondent | 20 |
| Centres without Phase 1 response | 2 (Dax, Nantes) |
| Patients included in sensitivity analysis | 104 |
| Centres included in mixed-effects model | 20 |

*Phase 1 (national survey) participation was defined as at least one physician respondent. Two centres were excluded from the mixed-effects sensitivity analysis.*

**Table S4. Sensitivity analysis of successful CRRT weaning using a mixed-effects multivariable logistic regression model with a random intercept for centre**

Outcome: successful CRRT weaning

Complete cases, n = 104. Centres Dax and Nantes were excluded due to the absence of Phase 1 survey responses.

| **Variable** | **Adjusted OR** | **95% CI** |
| --- | --- | --- |
| SAPS II (per 1 point) | 1.023 | 1.001–1.046 |
| Urine output on D-1 (per 100 mL) | 1.043 | 0.956–1.138 |
| Anuria (reason for CRRT initiation) | 0.427 | 0.201–0.983 |
| Reference serum creatinine (per µmol/L) | 0.999 | 0.991–1.008 |
| Random intercept (centre) | SD = 0.822 | — |
|  | Variance (σ²) = 0.675 | — |
|  | ICC = 0.17 | — |

*CRRT: continuous renal replacement therapy; D-1: day -1; ICC: intraclass correlation coefficient; SD: standard deviation*

*Fixed-effect estimates were consistent in direction and magnitude with the primary multivariable analysis, indicating that accounting for centre-level clustering did not materially influence the results.*

### **Table S5. Sensitivity analysis using a prespecified urine-output threshold**

**Outcome:** successful CRRT weaning
**Model:** multivariable logistic regression

| **Variable** | **Adjusted OR** | **95% CI** | **p-value** |
| --- | --- | --- | --- |
| SAPS II (per point) | 1.016 | 0.996 – 1.037 | 0.112 |
| Urine output on D−1 ≥500 mL/24h (vs <500 mL/24h) | 1.787 | 0.667 – 4.791 | 0.248 |
| **Anuria (reason for CRRT initiation)** | 0.299 | 0.115 – 0.780 | 0.014 |
| Reference serum creatinine (per µmol/L) | 0.998 | 0.991 – 1.004 | 0.525 |

*When urine output was dichotomised at a clinically plausible threshold, its association with successful weaning was no longer statistically significant; however, this sensitivity analysis did not materially alter the overall conclusions.*

**Table S6. Sensitivity analysis of successful CRRT weaning excluding constrained CRRT interruptions**

Outcome: successful CRRT weaning

Model: multivariable logistic regression

| **Variable** | **Adjusted OR** | **95% CI** | **p-value** |
| --- | --- | --- | --- |
| **SAPS II (per point)** | 1.017 | 0.989 – 1.046 | 0.237 |
| **Urine output on D-1 (per 100 mL)** | 0.993 | 0.914 – 1.078 | 0.863 |
| **Anuria (reason for CRRT initiation)** | 0.163 | 0.036 – 0.743 | 0.019 |
| **Reference serum creatinine (per µmol/L)** | 0.996 | 0.988 – 1.005 | 0.410 |

*Among the 116 patients initially included in the cohort, 16 CRRT cessation episodes were classified as constrained interruptions and excluded from this sensitivity analysis. These interruptions mainly reflected centre-specific organisational practices, including early switching to intermittent haemodialysis as a local routine, repeated circuit thrombosis or vascular access dysfunction precluding continuation of CRRT. Fixed-effect estimates were consistent in direction and magnitude with the primary multivariable model.*

**Table S7. Discrepancy between declared and observed CRRT weaning practices in septic AKI**

| Phase 1 (national survey: declared practices) | Phase 2 (multicentre retrospective cohort: observed practices) |
| --- | --- |
| CRRT weaning under vasopressor support is rarely considered acceptable. | 52% weaned under norepinephrine (median dose 0.22 µg/kg/min). |
| Declared urine output threshold: 612 ± 328 mL/day. | Median urine output 275 mL/day at D0; <200 mL/day during preceding days. |
| Fluid removal considered prerequisite before discontinuation. | 70% with positive fluid balance at weaning (median +5.0 L). |
| Net ultrafiltration reported as first-line fluid depletion strategy. | Net UF in ≤58% of patients at D−1. |
| Diuretics rarely used during CRRT by some clinicians. | Diuretics in 7% at D−1 and 26% at D0. |
| No formal weaning protocol in most centers. | Marked heterogeneity across centers. |

*Observed CRRT weaning practices frequently occur in the absence of clear renal recovery, under ongoing vasopressor support, and without effective fluid depletion, showing a marked discrepancy with clinicians’ declared thresholds and criteria reported in the Phase 1 (survey)*

**Survey on practices regarding weaning from continuous renal replacement therapy (CRRT) in septic acute kidney injury in the ICU**

Continuous renal replacement therapy (CRRT) remains widely used in cases of acute kidney injury (AKI) secondary to sepsis or septic shock requiring renal replacement therapy in the intensive care unit. While the initiation of RRT has been the focus of several high-quality studies, the process of weaning, particularly from CRRT, remains largely unexplored. Current practices are likely heterogeneous yet remain poorly characterized, and no specific guidelines exist. We therefore considered it important to investigate in greater detail the various approaches that intensivists, individually or collectively, may adopt when discontinuing continuous RRT.

**1. City and region of practice:**

_______________________________

**2. Age (years):**

- ☐ < 30 years
- ☐ 30-40 years
- ☐ 40-50 years
- ☐ 50-60 years
- ☐ > 60 years

**3. Experience in intensive care medicine (years):**

- ☐ < 5 years
- ☐ 5-10 years
- ☐ 10-20 years
- ☐ 20-30 years
- ☐ > 30 years

**4. Type of hospital and ICU you are currently working in:**

- ☐ University hospital (CHU)
- ☐ General hospital (CHG)
- ☐ Other type of hospital
- ☐ General ICU (polyvalent)
- ☐ Medical ICU
- ☐ Surgical ICU

**5. Do you hold a national or university diploma (DU/DIU) with specific training in renal replacement therapy?**

- ☐ Yes
- ☐ No

**6. Have you ever completed a specific training in RRT (outside a diploma, e.g. laboratory course, CME)?**

- ☐ Yes
- ☐ No

**7. Are you the physician in charge of RRT in your ICU?**

- ☐ Yes
- ☐ No

**8. Which modalities of RRT are available in your ICU?**

- ☐ Continuous RRT (CRRT) only
- ☐ Intermittent RRT only
- ☐ Both continuous and intermittent

**9. In your daily practice, how would you qualify your approach to CRRT weaning?**

- ☐ Early
- ☐ Late
- ☐ Intermediate

**10. In a patient with septic AKI requiring RRT, which factors lead you to prefer CRRT over intermittent therapy?**

- ☐ Hemodynamic instability
- ☐ Easier correction of acid-base disorders
- ☐ Reduced risk of osmotic shifts
- ☐ Facilitation of antibiotic use
- ☐ Staff trained only in CRRT
- ☐ Availability of CRRT in the unit
- ☐ Other (please specify)

**11. Do you sometimes stop CRRT before the end of the filter lifespan because you consider the patient no longer requires it?**

- ☐ Yes
- ☐ No

**12. Does the occurrence of adverse events (thrombocytopenia, metabolic issues, infection, catheter problem) lead you to reconsider stopping CRRT?**

- ☐ Yes
- ☐ No

**13. If yes, which adverse events?**

- ☐ Thrombocytopenia
- ☐ Hemorrhagic complications
- ☐ Citrate-related metabolic disorders
- ☐ Fever difficult to monitor
- ☐ Catheter-related infection
- ☐ Catheter dysfunction
- ☐ Other (please specify)

**14. Do you perform urinary biochemical analyses in patients on CRRT with preserved diuresis?**

- ☐ Yes
- ☐ No

**15. If yes, at what frequency and modality?**

- ☐ Occasionally (less than once per day)
- ☐ Daily
- ☐ Several times per day
- ☐ On spot urine samples
- ☐ On 24-hour urine collection

**16. If yes, which urinary parameters do you usually assess?**

- ☐ Urinary urea
- ☐ Urinary creatinine
- ☐ Urinary sodium
- ☐ Fractional excretion of urea
- ☐ Fractional excretion of sodium
- ☐ Calculated creatinine clearance
- ☐ Other (please specify)

**17. In an anuric patient on CRRT, does the occurrence of diuresis motivate you to consider weaning?**

- ☐ Yes
- ☐ No

**18. In an initially anuric patient, from what threshold of diuresis (ml/day) do you start considering CRRT weaning?**

**19. When using diuretics under CRRT for fluid depletion, in which situations do you initiate them?**

- ☐ Only in hemodynamically stable patients
- ☐ In patients still requiring vasopressors
- ☐ In anuric patients
- ☐ Only in patients with preserved/residual diuresis

**20. If you use diuretics for fluid depletion during CRRT, what is your strategy?**

- ☐ I do not use diuretics during CRRT
- ☐ I titrate progressively
- ☐ I prescribe a fixed daily dose
- ☐ I use loop diuretics
- ☐ I use thiazides
- ☐ Other (please specify)

**21. In a patient with anuric AKI, volume overload, and still on vasopressors (<0.5 µg/kg/min norepinephrine), what is your first-line approach for decongestion?**

- ☐ Initiate diuretics
- ☐ Initiate net ultrafiltration
- ☐ Combine diuretics and net ultrafiltration
- ☐ Neither diuretics nor net ultrafiltration

**22. Same scenario, but with preserved diuresis: what is your first-line approach?**

- ☐ Initiate diuretics
- ☐ Initiate net ultrafiltration
- ☐ Combine diuretics and net ultrafiltration
- ☐ Neither diuretics nor net ultrafiltration

**23. In a patient with anuric AKI, off vasopressors, what is your first-line approach for decongestion?**

- ☐ Initiate diuretics
- ☐ Initiate net ultrafiltration
- ☐ Combine diuretics and net ultrafiltration
- ☐ Neither diuretics nor net ultrafiltration

**24. Same scenario, but with preserved diuresis: what is your first-line approach?**

- ☐ Initiate diuretics
- ☐ Initiate net ultrafiltration
- ☐ Combine diuretics and net ultrafiltration
- ☐ Neither diuretics nor net ultrafiltration

**25. When performing ultrafiltration during CRRT, how do you determine the net UF rate?**

- ☐ Based on diuresis
- ☐ Based on clinical edema
- ☐ Based on imaging/echo signs of congestion
- ☐ Based on fluid balance
- ☐ Based on weight changes

**26. In an oliguric patient (300 ml/day), post-septic shock, recently off vasopressors, still fluid overloaded, how do you adjust net UF?**

- ☐ No limit, would reintroduce vasopressors if needed
- ☐ Reduce net UF if hemodynamically unstable
- ☐ Reduce net UF if urine output increases
- ☐ Increase net UF if urine output increases
- ☐ Increase net UF if edema persists
- ☐ Other (please specify)

**27. After metabolic recovery allowing CRRT discontinuation, do you sometimes continue CRRT only for net UF?**

- ☐ Always
- ☐ Often
- ☐ Occasionally
- ☐ Rarely
- ☐ Never

**28. How frequently do you attempt CRRT discontinuation?**

- ☐ Once a week
- ☐ Several times a week
- ☐ Daily
- ☐ Several times a day

**29. Do you have a local protocol for CRRT weaning in your ICU?**

- ☐ Yes
- ☐ No

**30. If yes, please describe briefly:**

**31. If not, do you think implementing a protocol would facilitate earlier weaning?**

- ☐ Yes
- ☐ No

**32. For which reasons do you first consider CRRT discontinuation?**

- ☐ End of filter session
- ☐ Serum biochemistry
- ☐ Urinary parameters
- ☐ Spontaneous diuresis recovery
- ☐ Response to diuretics
- ☐ Clinical stabilization
- ☐ Weight loss
- ☐ Other (please specify)

**33. When initiating weaning, do you switch from CRRT to intermittent RRT?**

- ☐ Yes (please specify)
- ☐ No

**34. If you switch to intermittent RRT, what are your main criteria?**

- ☐ Organizational/logistical reasons
- ☐ Hemodynamic stability
- ☐ Other (please specify)

**35. After initiating weaning, what criteria do you use to definitely discontinue CRRT?**

- ☐ Spontaneous diuresis recovery
- ☐ Response to diuretics
- ☐ Serum biochemistry
- ☐ Urinary biochemistry
- ☐ Hemodynamic/respiratory stability
- ☐ Resolution of edema/return to dry weight
- ☐ End of filter session
- ☐ Other (please specify)

**36. After CRRT discontinuation, what criteria lead you to restart RRT?**

- ☐ Severe/refractory metabolic disorders
- ☐ Clinical signs of volume overload
- ☐ Other (please specify)

**37. In your opinion, what defines a successful weaning from RRT?**

- ☐ No need to restart RRT for 2 days
- ☐ … for 3 days
- ☐ … for 7 days
- ☐ No need until ICU discharge
- ☐ Catheter removal possible
- ☐ Other (please specify)

**38. When do you usually remove the dialysis catheter during weaning?**

- ☐ Immediately after last CRRT session
- ☐ 24h after successful discontinuation
- ☐ 48h after
- ☐ 72h after
- ☐ At ICU discharge
- ☐ Other (please specify)

**39. In your opinion, can late discontinuation of CRRT be detrimental to renal recovery?**

- ☐ Yes
- ☐ No

**40. In your opinion, can early discontinuation of CRRT be detrimental to renal recovery?**

- ☐ Yes
- ☐ No

**41. Do you consider CRRT weaning differently in patients with chronic kidney disease?**

- ☐ Yes, earlier
- ☐ Yes, later
- ☐ No

**42. Does the cost of CRRT circuits influence your practice?**

- ☐ Yes
- ☐ Sometimes
- ☐ No

**43. Would you accept a protocol mandating CRRT discontinuation after stabilization, even without diuresis recovery, with the possibility to restart if needed?**

- ☐ Yes
- ☐ No

**44. If 'No', what are your reasons?**

- ☐ Criteria insufficient for first attempt
- ☐ Would require lactate clearance
- ☐ Would require complete vasopressor withdrawal
- ☐ Would require distance from last fluid resuscitation
- ☐ Other (please specify)

**45. At ICU discharge, do you arrange nephrology follow-up for patients who required CRRT?**

- ☐ Always
- ☐ Occasionally
- ☐ Never
- ☐ Post-ICU clinic in my ICU
- ☐ Referral to nephrologist
- ☐ No follow-up planned
